# Brain Injury Awareness Month — March 2014

**Published:** 2014-03-14

March is Brain Injury Awareness Month. Through scientific research, programs, and education, CDC works to prevent traumatic brain injury (TBI) from all causes and ensure that persons with a TBI receive optimal care. Whether occurring from a fall in the home or on a playground, in sports, in a car crash, or by being struck by an object or another person, a TBI from any cause can disrupt the normal functions of the brain and can range in severity from a mild concussion to a severe, life-threatening injury. Most TBIs can be prevented.

In 2010, in the United States, 2.5 million emergency department visits, hospitalizations, or deaths were associated with TBI, either alone or with other injuries or illnesses. Additionally, research indicates that men in the United States have higher rates of TBI than women. The very young and older adults also have higher rates of TBI resulting from falls. Adults aged ≥65 years have the highest rates of TBI-related hospitalization and are more likely to die from a TBI (either TBI alone or with other injuries or illnesses) than any other age group. Additionally, adolescents and young adults (i.e., persons aged 15–24 years) have the highest rates of motor vehicle–related TBIs.

The burden of TBI can be reduced through primary prevention strategies and improvements in the health and quality of life for persons living with a TBI. CDC focuses on integrating public health prevention and health-care delivery systems, including efficient, effective care and rehabilitation services to address the issue of TBI among at-risk populations. Strategies such as buckling children in age- and size-appropriate car seats and starting a regular exercise program to reduce older adult falls are effective ways to reduce the incidence of TBI.Persons with a suspected TBI should receive medical care. Additional information about TBI and its management is available at http://www.cdc.gov/traumaticbraininjury, information about preventing motor vehicle-related TBIs is available at http://www.cdc.gov/motorvehiclesafety, and information about preventing fall-related TBIs is available at http://www.cdc.gov/homeandrecreationalsafety/falls.

